# Diagnostic Accuracy of Chest X-ray Computer-Aided Detection Software for Detection of Prevalent and Incident Tuberculosis in Household Contacts

**DOI:** 10.1093/cid/ciae528

**Published:** 2024-12-18

**Authors:** Liana Macpherson, Sandra V Kik, Matteo Quartagno, Francisco Lakay, Marche Jaftha, Nombuso Yende, Shireen Galant, Saalikha Aziz, Remy Daroowala, Richard Court, Arshad Taliep, Keboile Serole, Rene T Goliath, Nashreen Omar Davies, Amanda Jackson, Emily Douglass, Bianca Sossen, Sandra Mukasa, Friedrich Thienemann, Taeksun Song, Morten Ruhwald, Robert J Wilkinson, Anna K Coussens, Hanif Esmail, Imaging of TB Household Contacts Group, Clifton E Barry, Jerrold J Ellner, JoAnne L Flynn, Torben Heinsohn, C Robert Horsburgh, Karen R Jacobson, Stephanus T Malherbe, Padmini Salgame, Dylan Sheerin, Elizabeth Streicher, Mpho Tlala, Laura E Via, Gerhard Walzl, Robin Warren, James Warwick

**Affiliations:** MRC Clinical Trials Unit, University College London, London, United Kingdom; FIND, Geneva, Switzerland; MRC Clinical Trials Unit, University College London, London, United Kingdom; Centre for Infectious Diseases Research in Africa, Institute of Infectious Disease and Molecular Medicine and Department of Medicine, University of Cape Town, Cape Town, South Africa; Institute of Infectious Disease and Molecular Medicine and Department of Pathology, University of Cape Town, Cape Town, South Africa; Institute of Infectious Disease and Molecular Medicine and Department of Pathology, University of Cape Town, Cape Town, South Africa; Institute of Infectious Disease and Molecular Medicine and Department of Pathology, University of Cape Town, Cape Town, South Africa; Centre for Infectious Diseases Research in Africa, Institute of Infectious Disease and Molecular Medicine and Department of Medicine, University of Cape Town, Cape Town, South Africa; Centre for Infectious Diseases Research in Africa, Institute of Infectious Disease and Molecular Medicine and Department of Medicine, University of Cape Town, Cape Town, South Africa; Centre for Infectious Diseases Research in Africa, Institute of Infectious Disease and Molecular Medicine and Department of Medicine, University of Cape Town, Cape Town, South Africa; Centre for Infectious Diseases Research in Africa, Institute of Infectious Disease and Molecular Medicine and Department of Medicine, University of Cape Town, Cape Town, South Africa; Centre for Infectious Diseases Research in Africa, Institute of Infectious Disease and Molecular Medicine and Department of Medicine, University of Cape Town, Cape Town, South Africa; Centre for Infectious Diseases Research in Africa, Institute of Infectious Disease and Molecular Medicine and Department of Medicine, University of Cape Town, Cape Town, South Africa; Centre for Infectious Diseases Research in Africa, Institute of Infectious Disease and Molecular Medicine and Department of Medicine, University of Cape Town, Cape Town, South Africa; Centre for Infectious Diseases Research in Africa, Institute of Infectious Disease and Molecular Medicine and Department of Medicine, University of Cape Town, Cape Town, South Africa; Rutgers–New Jersey Medical School, Center for Emerging Pathogens, Newark, New Jersey, USA; Centre for Infectious Diseases Research in Africa, Institute of Infectious Disease and Molecular Medicine and Department of Medicine, University of Cape Town, Cape Town, South Africa; Centre for Infectious Diseases Research in Africa, Institute of Infectious Disease and Molecular Medicine and Department of Medicine, University of Cape Town, Cape Town, South Africa; Centre for Infectious Diseases Research in Africa, Institute of Infectious Disease and Molecular Medicine and Department of Medicine, University of Cape Town, Cape Town, South Africa; Department of Internal Medicine, University Hospital of Zurich, University of Zurich, Zurich, Switzerland; Institute of Infectious Disease and Molecular Medicine and Department of Pathology, University of Cape Town, Cape Town, South Africa; FIND, Geneva, Switzerland; Centre for Infectious Diseases Research in Africa, Institute of Infectious Disease and Molecular Medicine and Department of Medicine, University of Cape Town, Cape Town, South Africa; Francis Crick Institute, London, United Kingdom; Department of Infectious Diseases, Imperial College London, London, United Kingdom; Centre for Infectious Diseases Research in Africa, Institute of Infectious Disease and Molecular Medicine and Department of Medicine, University of Cape Town, Cape Town, South Africa; Infectious Diseases and Immune Defence Division, Walter and Eliza Hall Institute of Medical Research (WEHI), Parkville, Australia; MRC Clinical Trials Unit, University College London, London, United Kingdom; Centre for Infectious Diseases Research in Africa, Institute of Infectious Disease and Molecular Medicine and Department of Medicine, University of Cape Town, Cape Town, South Africa; WHO Collaborating Centre for TB Research and Innovation, Institute for Global Health, University College London, London, United Kingdom

**Keywords:** tuberculosis, asymptomatic, computer-aided detection, chest X-ray, active case finding

## Abstract

**Background:**

World Health Organization (WHO) tuberculosis (TB) screening guidelines recommend computer-aided detection (CAD) software for chest radiograph (CXR) interpretation. However, studies evaluating their diagnostic and prognostic accuracy are limited.

**Methods:**

We conducted a prospective cohort study of household contacts of rifampicin-resistant TB in South Africa. Participants underwent baseline CXR and sputum investigation (routine [single spontaneous] and enhanced [additionally 2–3 induced]) for prevalent TB and follow-up for incident TB. Three CXR-CAD software products (CAD4TBv7.0, qXRv3.0.0, and Lunit INSIGHT v3.1.4.111) were compared. We evaluated their performance to detect routine and enhanced prevalent and incident TB, comparing performance with blood tests (Xpert MTB host-response, erythrocyte sedimentation rate, C-reactive protein, QuantiFERON) in a subgroup.

**Results:**

483 participants were followed up for 4.6 years (median). There were 23 prevalent (7 routinely diagnosed) and 38 incident TB cases. The AUC ROCs (95% CIs) to identify prevalent TB for CAD4TBv7.0, qXRv3.0.0, and Lunit INSIGHT v3.1.4.111 were .87 (.77–.96), .88 (.79–.97), and .91 (.83–.99), respectively. More than 30% with scores above recommended CAD thresholds who were bacteriologically negative on routine baseline sputum were subsequently diagnosed by enhanced sputum investigation or during follow-up. The AUC performance of baseline CAD to identify incident cases ranged between .60 and .65. Diagnostic performance of CAD for prevalent TB was superior to blood testing.

**Conclusions:**

Our findings suggest that the potential of CAD-CXR screening for TB is not maximized as a high proportion of those above current thresholds, but with a negative routine confirmatory sputum, have true TB disease that may benefit intervention.

## Teaser

CAD-CXR had high diagnostic accuracy for prevalent tuberculosis in household tuberculosis contacts. However, >30% with scores above recommended CAD thresholds who were bacteriologically negative on baseline routine testing were subsequently diagnosed with tuberculosis suggesting the potential of CAD-CXR screening is not maximized.

The World Health Organization (WHO) estimated that there are up to 2.74 million people with undiagnosed tuberculosis (TB) [1]. Chest X-ray (CXR) screening is recognized as the most effective approach to proactively identify undiagnosed TB cases, as 50% are symptom screen negative [2–9]. In 2021, the WHO screening guidelines endorsed computer-aided detection (CAD) software for automated radiographic interpretation [8].

Computer-aided detection software uses trained deep-learning algorithms to interpret CXR for signs of TB. Several studies have reported accuracy equivalent to human readers [8, 10–12]. However, high-quality prospective studies evaluating the diagnostic accuracy in the screening setting are limited [13, 14].

Confirmatory bacteriological testing is often done by assessment of a single, spontaneously produced sputum sample with molecular detection of *Mycobacterium tuberculosis* (*Mtb*) DNA (eg, Xpert MTB/RIF) [8]. This approach is insensitive as not all those with a positive triage test are able to expectorate sputum for confirmatory testing [15]. Bacteriological confirmation increases with multiple samples, sputum induction, and/or use of culture, but such enhanced measures are not typically performed routinely.

A recent meta-analysis demonstrated that those with CXR abnormalities suggestive of TB without sputum bacteriological confirmation have a 10% risk per year of being diagnosed with bacteriologically positive TB [16]. However, the majority of the contributory studies were historical, pre–human immunodeficiency virus (pre-HIV) epidemic, and used conventional CXR, and no contemporary studies have evaluated this risk utilizing digital CXR and CAD approaches.

Blood tests predictive of future TB risk are a priority for development and could potentially be used alongside CXR screening to identify individuals with future TB risk. However, the evaluation of such tests in a screening population alongside or in combination with CXR has not previously been undertaken.

In this study we screened household contacts (HHCs) of patients with rifampicin-resistant (RR) TB by digital CXR and undertook long-term follow-up for development of TB disease in the absence of preventive therapy. Our aims were as follows: (1) evaluate the diagnostic accuracy of 3 CAD software packages against microbiological reference standards to detect prevalent and incident pulmonary TB, (2) determine the sensitivity and specificity of CAD software using the recommended threshold scores, and (3) compare and combine CAD scores with blood testing to detect prevalent and incident TB.

## METHODS

The study was conducted in Khayelitsha, South Africa. Recruitment took place between November 2014 and September 2017 with follow-up until May 2021 [17]. Ethical approval was received from University of Cape Town (449/2014), Boston University (H-35831), Rutgers University (Pro2018001966), the National Institutes of Health (NIH) (DMID 16-0112), and University College London (19219/001).

Eligible individuals were HHCs of RR-TB aged 18 years or older. At screening, participants underwent medical history, physical examination, HIV testing, TB symptoms screen (unexplained cough for ≥2 weeks, fever, night sweats, and weight loss), and digital CXR. Participants were investigated for TB at baseline regardless of symptoms, with 1 attempted spontaneous spot sputum sample followed by 2–3 induced sputum samples for a total of 3 samples. Sputum induction was by nebulization of 3% hypertonic saline. Samples were processed in the accredited laboratories with auramine sputum smear, Xpert MTB/RIF, and mycobacterial growth indicator tube (MGIT) culture. Participants of child-bearing potential underwent urinary pregnancy testing and, if positive, were excluded from the study.

Posterior-anterior CXRs were performed using a digital X-ray machine (Phillips Essenta DR). All CXRs were reported by a medical officer blinded to clinical details and microbiological outcomes. Three CAD software products were evaluated: CAD4TB version 7.0 (CAD4TBv7; Delft Imaging, ’s-Hertogenbosch, Netherlands), qXR version 3.0.0 (qXRv3; Qure.ai, Mumbai, India), and Lunit INSIGHT CXR version 3.1.4.111 (Lunitv3; Lunit, Seoul, South Korea). For analysis, for qXRv3 and Lunitv3 the manufacturer-recommended threshold scores of 0.5 and 0.15, respectively, were used and for CAD4TBv7, where the manufacturer does not specify a threshold, a commonly used threshold of 50 was used (see Supplementary Methods).

As part of a nested study, participants, who were HIV-uninfected and asymptomatic, additionally consented to also undergo blood sampling for a biomarker substudy [17]. Blood samples were taken for C-reactive protein (CRP), erythrocyte sedimentation rate (ESR), QuantiFERON Gold-in-tube (QFT-Gold; Qiagen), and Tempus Tubes (Applied Biosystems), stored at −80°C to preserve blood RNA for transcriptomic analysis including the 3-gene RNA Xpert MTB Host Response (MTB-HR; Cepheid, Sunnyvale, CA, USA) (see Supplementary Methods).

No participants received preventive therapy as they were contacts of RR-TB in line with national and some international guidelines at the time of the study [18]. Participants who were bacteriologically positive or with clinical concern of TB were referred to the statutory TB clinic where the decision to start TB treatment was determined.

Participants were asked to attend the clinic for assessment if they developed TB symptoms. All participants were then invited for systematic re-screening between 24 and 36 months, irrespective of symptoms, with 3 sputum samples taken (induced if needed) sent for smear, Xpert MTB/RIF, and culture. No repeat CXR was conducted at re-screening. To ensure that all episodes of treated TB within the Western Cape Province were captured, participants consented to access to their health service records via the Provincial Health Data Centre (PHDC) (which included all results from National Health Laboratory Service and clinic prescriptions) [19]. This search took place on 19 May 2021.

A case of prevalent TB was defined as at least 1 baseline sputum sample culture and/or Xpert MTB/RIF positive for *Mtb* where the participant was treated for TB. We created subgroups of participants with prevalent disease to reflect cases who would be identified through routine screening (routine prevalent) and those who would only be identified through more intensive investigation (enhanced prevalent). Routine prevalent cases were defined as those cases initiated on TB treatment in whom *Mtb* was detected using Xpert MTB/RIF on the first single spontaneously produced sputum sample [20]. Enhanced prevalent cases were defined as those initiated on TB treatment in whom *Mtb* was detected in any other baseline sputum sample by either Xpert MTB/RIF or culture. Incident TB was defined as cases initiated on TB treatment in whom at least 1 follow-up sputum sample culture and/or Xpert MTB/RIF was *Mtb* positive or where a clinician independent of the study made a clinical decision to start TB treatment. We classified participants as not having TB if all baseline and follow-up samples were negative for *Mtb*, and they were not initiated on TB treatment.

Sample size was determined by the parent study [17]. The area under the receiver operating characteristic (AUC ROC) curve was calculated to evaluate diagnostic performance. Biomarkers were included in the linear models as covariates. We used the Delong test for differences in the AUC of the ROC curves. We calculated the sensitivity and specificity of CAD software using the manufacturer’s prespecified or commonly used thresholds. Analyses were also performed for several prespecified subgroups: people with HIV (PWH), those with a history of previous TB, and smokers. Participants were excluded from analysis if any one of the CAD software products did not provide a score due to error, or if data were incomplete for the baseline sputum results. For the biomarker analysis, participants with missing data were excluded from each model and we then performed a sensitivity analysis excluding all cases with any missing biomarker data. Statistical analyses were conducted with R version 4.0.5 (2021-03-31) (R Foundation for Statistical Computing, Vienna, Austria).

## RESULTS

**Figure 1.**
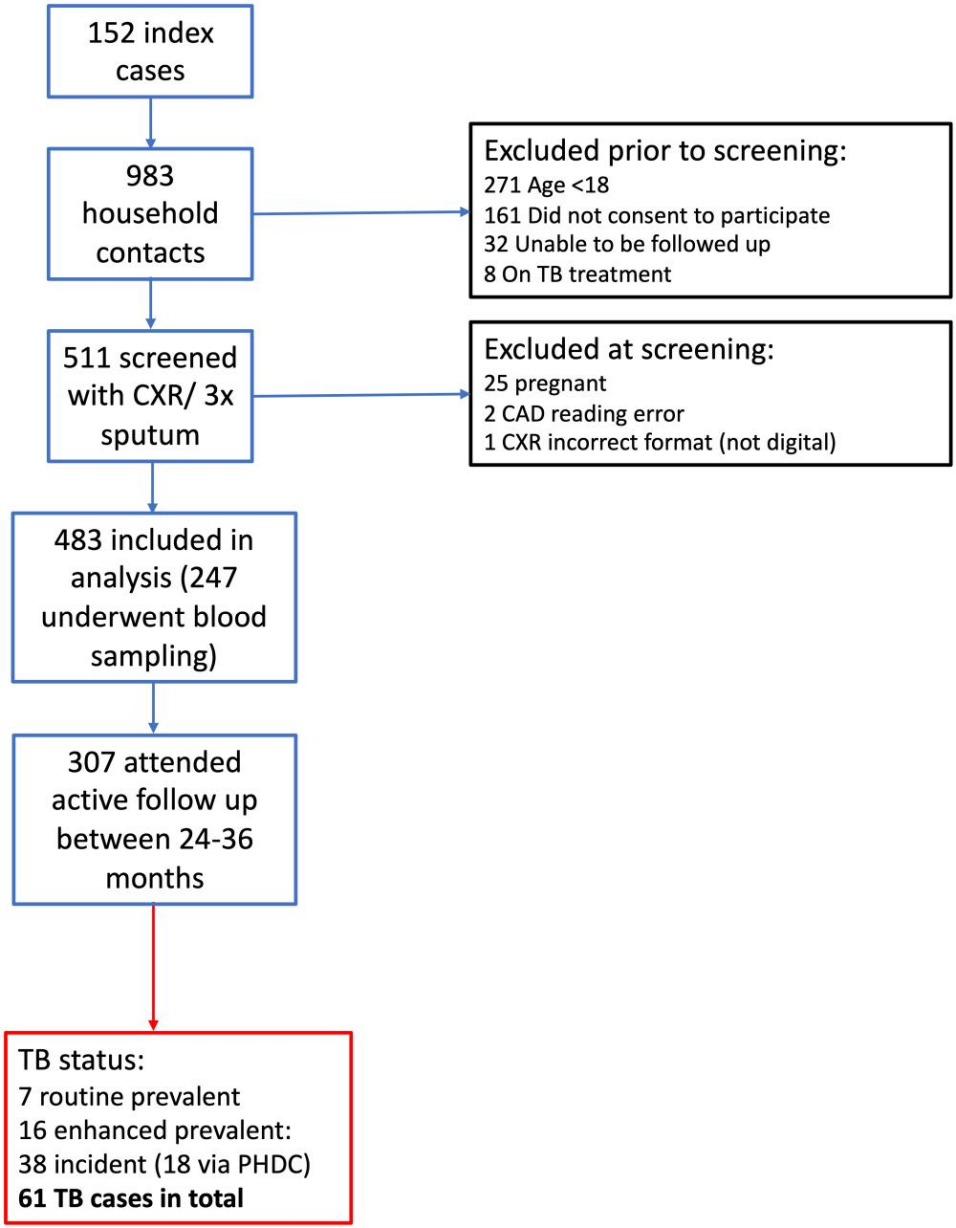
Study population. Details of participants who were included in this analysis including a description of TB disease status. Abbreviations: CAD, computer-aided detection; CXR, chest X-ray; PHDC, Provincial Health Data Centre; TB, tuberculosis.

A total of 983 HHCs of participants with RR-TB were identified; 263 were younger than 18 years old, 161 did not consent, 32 were unable to be followed up, and 8 were already on TB treatment, leaving 511 eligible adult participants who provided consent. A total of 483 participants were included in the analysis; exclusions included pregnancy (n = 25) and no CAD reading (n = 3) (Figure 1). The median age was 33 years (interquartile range [IQR], 26–47 years), 308 (61%) were female, 109 (23%) had a history of previous TB, and 136 (28%) were PWH (Table 1). For those PWH, 62.5% were on antiretroviral therapy (ART) and a recent CD4 count was available for 54 participants (median, 413.5 cells/mm^3^; IQR, 236–562 cells/mm^3^). The participants were followed up for a median of 4.6 years (IQR, 3.9–5.2 years). A total of 307 of 483 (64%) were rescreened with sputum sampling at 24–36 months (154 unable to attend/uncontactable, 16 deaths, and 6 unable to produce sample). A total of 467 of 483 (97%) had outcomes and results were available through the PHDC database. Two hundred forty-seven HIV-uninfected participants without TB symptoms met the inclusion criteria to undergo biomarker blood sampling.

**Table 1.** Baseline Characteristics

	All Participants (n = 483)	Participants With Routine Prevalent TB (n = 7)	Participants With Enhanced Prevalent TB (n = 16)	Participants With Incident TB (n = 38)	Participants With no TB Diagnosis (n = 422)
Age, median (IQR), y	33 (26–47)	40 (38–53)	38 (29–48)	36 (29–48)	33 (26–47)
Sex, female, n (%)	308 (64%)	2 (29%)	10 (63%)	26 (68%)	270 (64%)
Baseline symptoms present (yes), n (%)	51 (11%)	4 (57%)	4 (25%)	6 (16%)	37 (9%)
Cough >2/52	32 (7%)	3 (43%)	2 (13%)	5 (13%)	22 (5%)
Night sweats	23 (5%)	4 (57%)	0	4 (11%)	15 (4%)
Weight loss	28 (6%)	2 (29%)	4 (25%)	3 (8%)	19 (5%)
Fever	2 (0.5%)	0	0	1 (3%)	1 (0.2%)
Previous history of TB, n (%)	109 (23%)	3 (43%)	5 (31%)	12 (32%)	89 (21%)
HIV infected, n (%)	136 (28%)	4 (57%)	7 (44%)	18 (47%)	107 (25%)
Previous smoker, n (%)	164 (34%)	3 (43%)	5 (31%)	18 (47%)	138 (33%)
BMI, median (IQR), kg/m^2^	28 (22–33)	24 (21–25)	24 (19–29)	24 (20–32)	28 (23–33)
Proportion CXR consistent with TB—determined by human reader, n (%)	74 (15%)	5 (71%)	9 (56%)	9 (24%)	51 (12%)
Proportion able to produce spontaneous sputum sample at baseline, n (%)	292 (60%)	7 (100%)	9 (56%)	21 (55%)	255 (60%)
Proportion able to produce sputum sample with induction at baseline, n (%)	466 (96%)	7 (100%)	15 (94%)	37 (97%)	406 (96%)
Baseline sputum samples, n (%)					
None	5 (1%)	0	1 (6%)	0	5 (1%)
1	45 (9%)	0	1 (6%)	3 (8%)	41 (10%)
2	17 (4%)	0	13 (81%)	4 (11%)	12 (3%)
3	406 (84%)	7 (100%)	1 (6%)	30 (79%)	355 (84%)
Missing	10 (2%)	0	0	1 (3%)	9 (2%)
CAD4TB score, median (IQR)	8.7 (3.7–23.9)	75 (52.1–79.5)	57.3 (32.4–67.4)	11.7 (4.7–33.6)	7.7 (3.6–19)
qXR score, median (IQR)	0.01 (0.01–0.03)	0.88 (0.49–0.91)	0.44 (0.23–0.75)	0.16 (0.01–0.21)	0.01 (0.01–0.02)
Lunit INSIGHT CXR score, median (IQR)	1.5 (0.9–4)	80.6 (75.1–96.4)	67.9 (49.6–96.5)	3.2 (1.4–32.9)	1.4 (0.9–2.9)

Detailing the participants’ demographic data, sputum samples, and scores from the CAD software for baseline chest radiograph interpretation. This information is displayed for all participants, those with routinely diagnosed prevalent TB, enhanced prevalent TB, and incident TB.

Abbreviations: BMI, body mass index; CAD, computer-aided detection; CAD4TB, CAD4TB version 7; CXR, chest X-ray; HIV, human immunodeficiency virus; IQR, interquartile range; Lunit INSIGHT CXR, Lunit INSIGHT CXR version 3.1.4.111; qXR, qXR version 3.0.0; TB, tuberculosis.

A total of 292 of 483 participants (60%) produced a baseline spontaneous sputum sample, 466 (96%) produced an induced sample (Table 1). Twenty-three participants (4.7%) had bacteriologically confirmed TB infection at baseline (prevalent TB); 7 (30%) were routine prevalent cases and 16 were enhanced prevalent cases.

Of the 460 participants not diagnosed with prevalent TB, a further 38 (8.3%) cases of incident TB (21 captured via PHDC) were identified during follow-up. The median time to diagnosis was 24 months (IQR, 10–34 months). Of the 38 incident cases, 10 (34%) were diagnosed within 12 months (8 via PHDC), 7 (16%) between 13 and 24 months (5 via PHDC), 11 (29%) between 25 and 36 months (3 via PHDC), and 7 (18%) between 37 and 63 months (5 via PHDC). Six incident cases were clinically diagnosed, but not bacteriologically confirmed (5 via PHDC). Fourteen of 55 bacteriologically confirmed cases (4/23 prevalent and 10/32 incident) were RR.

Fifty-one (11%) participants reported at least 1 TB symptom at baseline; of these, 8 were confirmed TB at baseline (35% of the 23 prevalent cases). Of the 23 prevalent cases, 8 (35%) were smear positive, 14 (61%) were Xpert MTB/RIF positive, and 20 (87%) were culture positive in baseline samples (Supplementary Table 1). Of the 38 incident cases, 79% reported TB symptoms at the time of diagnosis and 3 had extrapulmonary disease. All 38 incident cases were included in the CAD diagnostic accuracy analysis.

**Figure 2.**
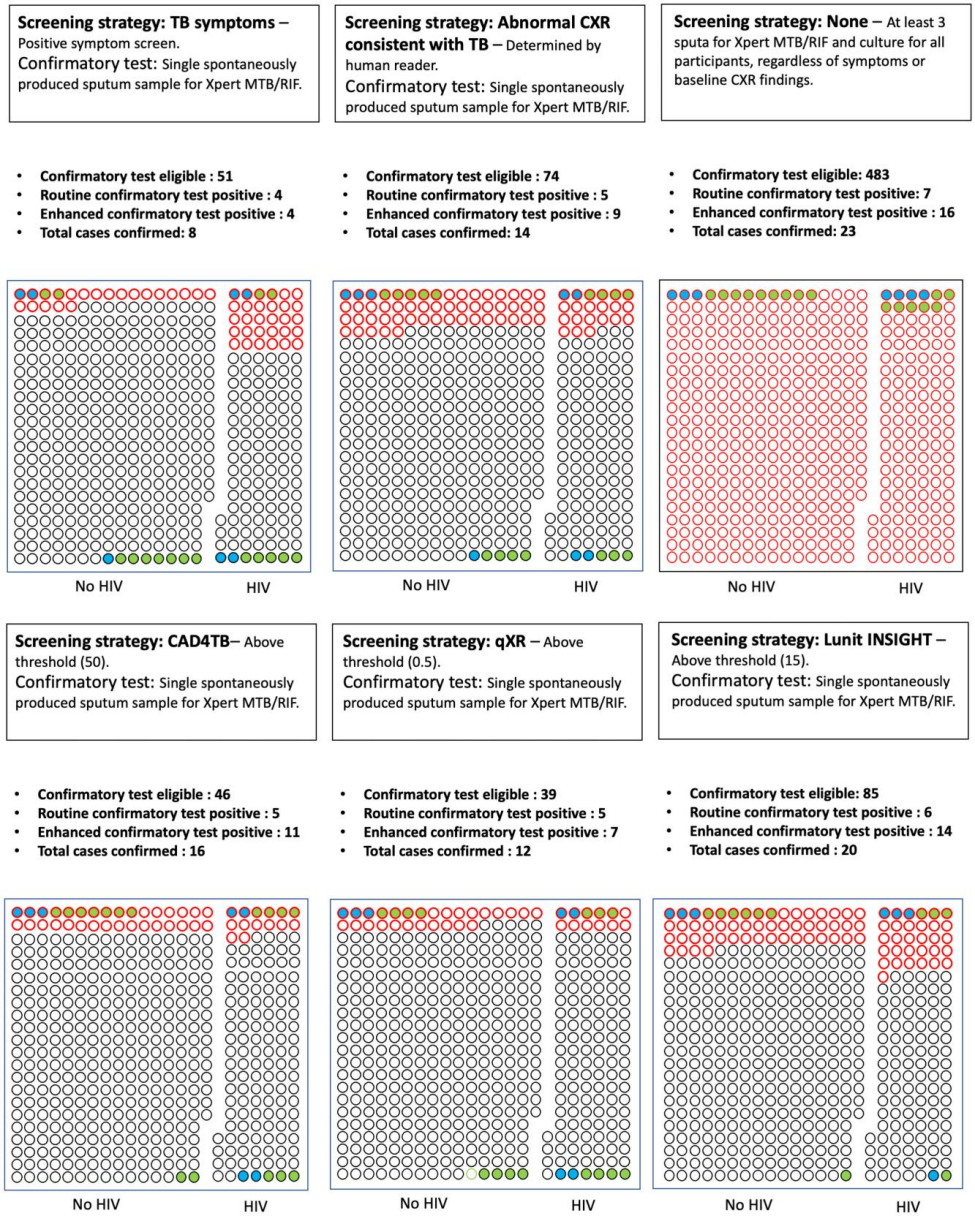
Yields of TB-screening strategies. In this representation of the yields of these screenings, each dot represents 1 study participant. The color coding of the participants represents TB disease status as defined by bacteriological investigation. Participants have also been grouped by HIV status to show the relative representation of TB in each population. The figure is accompanied by a table, which shows the sensitivity and specificity of the different screening approaches (the numbers represent proportions and 95% CIs). Abbreviations: CAD4TB, CAD4TB version 7; CXR, chest-X-ray; HIV, human immunodeficiency virus; Lunit INSIGHT CXR, Lunit INSIGHT CXR version 3.1.4.111; qXR, qXR version 3.0.0; TB, tuberculosis.

We explored the yields of the following hypothetical screening strategies: (1) positive symptom screen followed by Xpert MTB/RIF on a spontaneously produced sputum sample and (2) positive CXR (human read) followed by Xpert MTB/RIF on a spontaneously produced sputum sample. These strategies detected 4 of 23 (17%) and 5 of 23 (22%) of the prevalent TB cases in this cohort, respectively (Figure 2).

**Figure 3.**
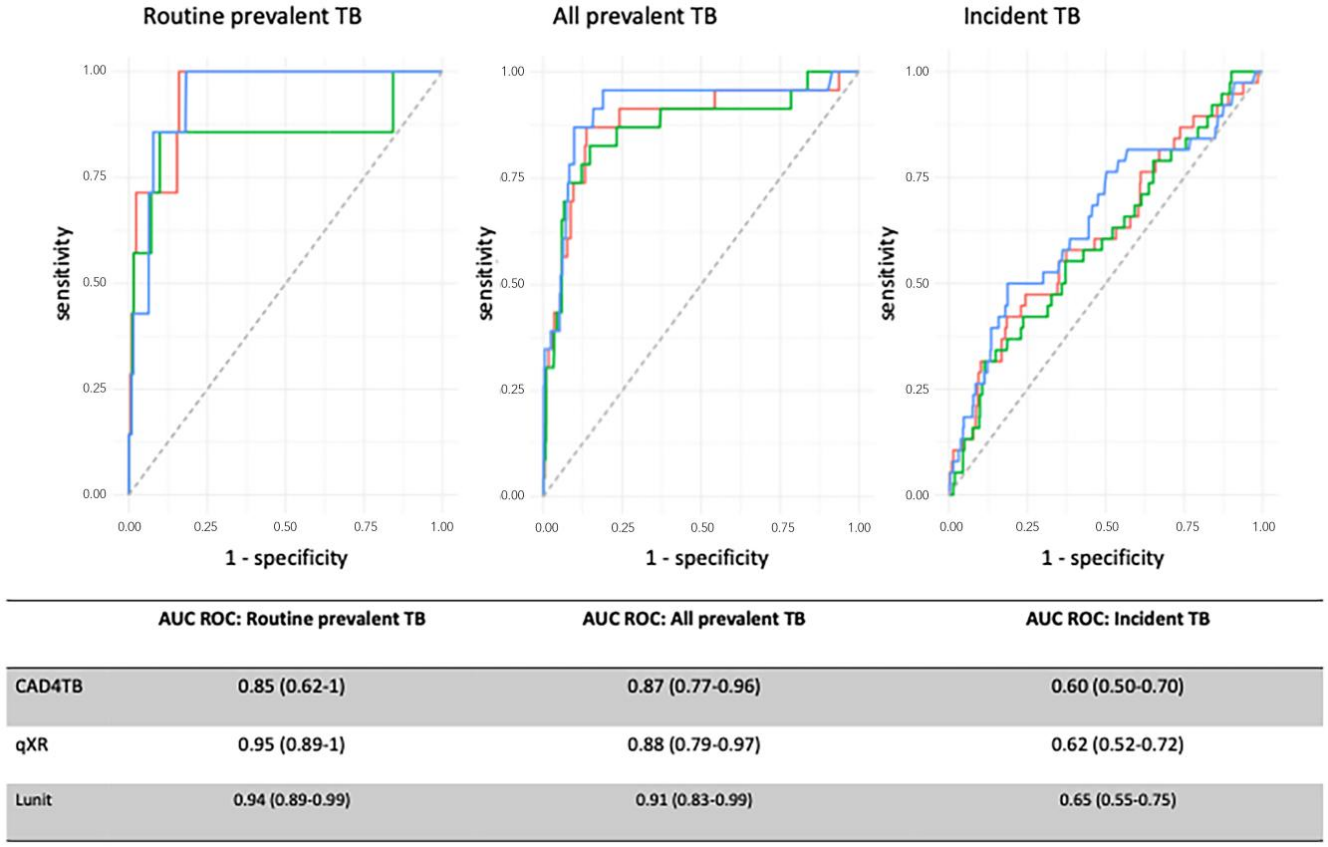
Performance of 3 different CAD software products for the detection of prevalent and incident TB cases. The CAD software products evaluated were CAD4TB version 7.0 (CAD4TB; Delft Imaging, ‘s-Hertogenbosch, Netherlands), qXR version 3.0.0 (qXR; Qure.ai, Mumbai, India), and Lunit INSIGHT CXR version 3.1.4.111 (Lunit; Lunit, Seoul, South Korea). Routine prevalent TB was defined as those initiated on TB treatment in whom *Mycobacterium tuberculosis (Mtb*) was detected using Xpert MTB/RIF on the first single, spontaneously produced baseline sputum sample; all prevalent TB was defined as those initiated on TB treatment in whom *Mtb* was detected using Xpert MTB/RIF and/or culture on any other baseline sputum sample; and incident TB was defined as those cases initiated on TB treatment in whom *Mtb* was detected in at least 1 follow-up sputum sample by Xpert MTB/RIF and/or culture or where the initiation of TB treatment was based on clinical grounds. Accuracy was measured using the AUC ROC curve. The numbers represent proportions (95% CI). Abbreviations: AUC ROC, area under the receiver operating characteristic; CAD, computer-aided detection; CAD4TB, CAD4TB version 7; Lunit INSIGHT CXR, Lunit INSIGHT CXR version 3.1.4.111; qXR, qXR version 3.0.0; TB, tuberculosis.

All CAD software were effective in identifying prevalent TB, with no statistically significant differences. The AUCs were .85 (95% CI, .62–1) to .95 (95% CI, .89–1) for identifying routine prevalent and .87 (95% CI, .77–.96) to .91 (95% CI, .83–.99) for identifying all prevalent cases (Figure 3). The CAD software performed less well at identifying participants with incident TB on baseline CXR, with AUCs of .60 (95% CI, .50–.70) to .65 (95% CI, .55–.75) (sensitivity analysis restricting to 32 microbiologically confirmed cases: AUC, .58 [.47–.69] to .63 [.52–.74]). There was no substantial difference in the accuracy to detect TB occurring between 1 and 12 months, 13 and 24 months, or over 24 months after study entry (Supplementary Figure 1).

All CAD systems detected prevalent TB with significantly greater accuracy in participants with no history of previous TB (*P* = .02, .04, and .01). There was also a trend of better performance in those who did not have HIV (*P* = .09, .18, and .5) (see Supplementary Table 2).

Using the manufacturer-recommended thresholds, the sensitivity/specificity to detect routine prevalent TB cases was 0.71/0.91 for CAD4TBv7, 0.72/0.93 for qXRv3, and 0.86/0.83 for Lunitv3. The sensitivity/specificity to detect all prevalent cases was 0.7/0.93 for CAD4TBv7.0, 0.57/0.94 for qXRv3, and 0.87/0.86 for Lunitv3 (Figure 5 and Supplementary Table 3). By comparison, the sensitivity/specificity for the human reader to detect routine prevalent or all prevalent cases was 0.71/0.85 and 0.61/0.87, respectively (Figure 2).

**Figure 4.**
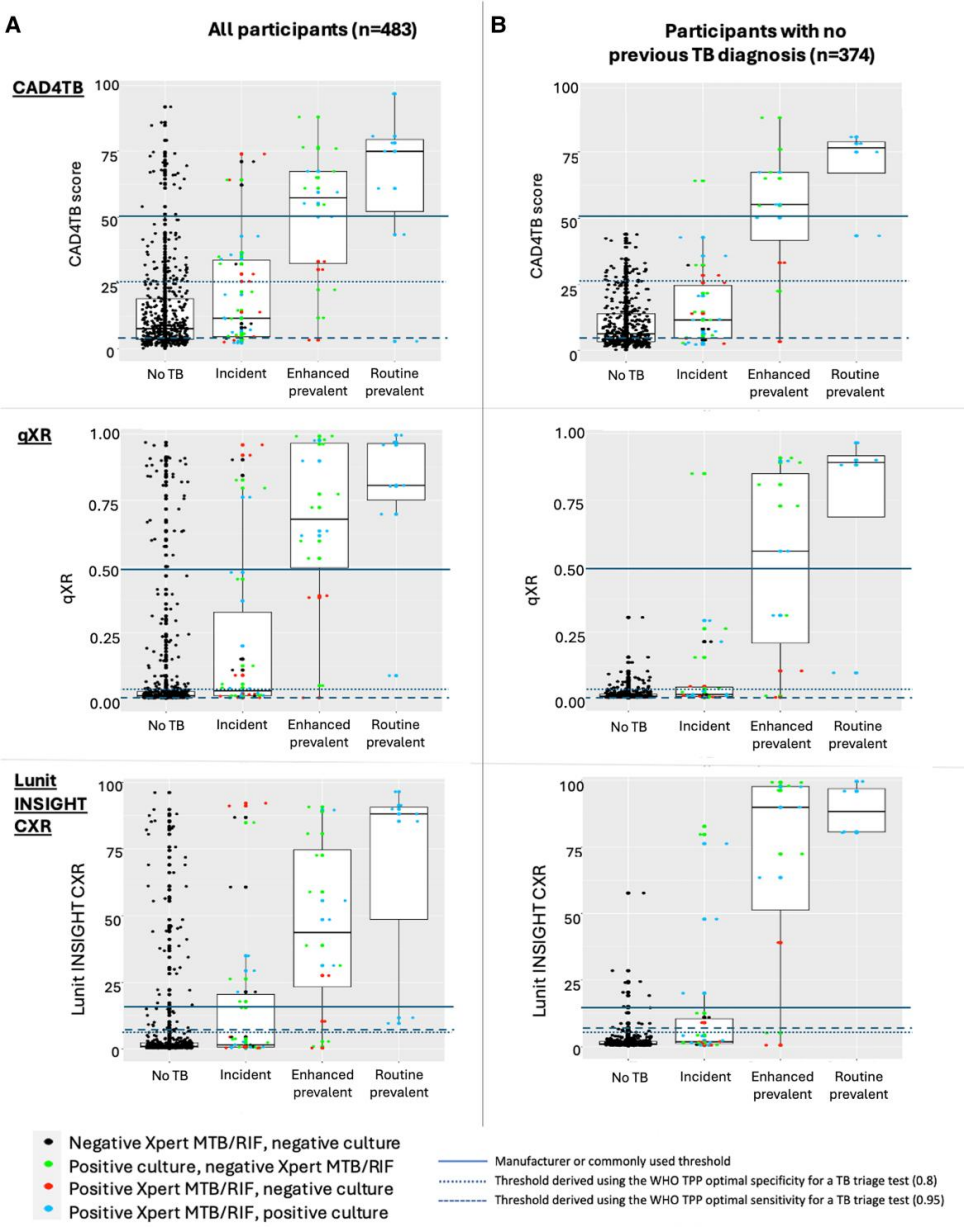
Representation of study participants above and below the manufacturer’s or commonly used threshold for each CAD software (CAD4TB version 7 [CAD4TB], qXR version 3.0.0 [qXR], and Lunit INSIGHT CXR version 3.1.4.111 [Lunit]) by TB status. The figure provides a visual representation of the distribution of CAD software scores for each software used (CAD4TB, qXR, Lunit), broken down by TB disease status. Panels under (A) include all 483 participants and panels under (B) include the 374 participants without a previous history of TB. Routine prevalent TB was defined as those initiated on TB treatment in whom *Mycobacterium tuberculosis* (*Mtb*) was detected using Xpert MTB/RIF on the first single, spontaneously produced baseline sputum sample; enhanced prevalent TB was defined as those initiated on TB treatment in whom *Mtb* was detected using Xpert MTB/RIF and/or culture on any other baseline sputum sample not included as routine prevalent; and incident TB was defined as those cases initiated on TB treatment in whom *Mtb* was detected in at least 1 follow-up sputum sample by Xpert MTB/RIF and/or culture or where the initiation of TB treatment was based on clinical grounds. Each dot represents a study participant, and the color coding of the dot represents the bacteriological status of that participant. For example, some incident cases are represented by black dots, indicating that these participants were bacteriologically negative; these represent the cases of incident TB that were diagnosed clinically. Horizontal lines have been added to represent 3 thresholds—the threshold above which the score is consistent with TB infection as recommended by the manufacturer or commonly used in practice, the threshold derived by using the WHO TPP optimal specificity for a TB triage test, and the threshold derived by using the WHO TPP optimal sensitivity for a TB triage test. The latter 2 thresholds were generated using all study participants/all prevalent cases of TB. Abbreviations: CAD, computer-aided detection; Lunit INSIGHT CXR, Lunit INSIGHT CXR version 3.1.4.111; qXR, qXR version 3.0.0; TB, tuberculosis; TPP, target product profile; WHO TPP, world health organization target product profile.

**Figure 5.**
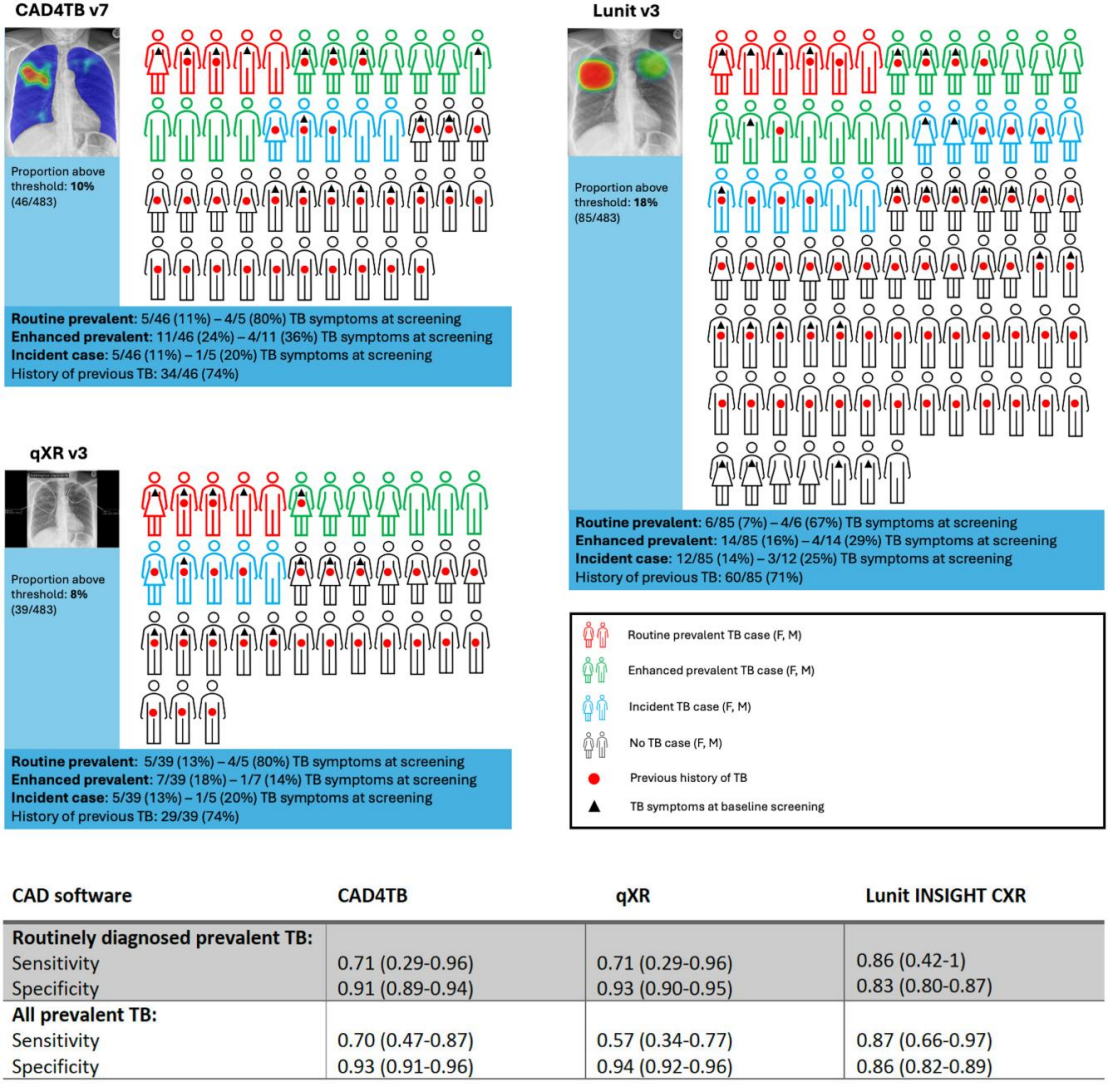
Representation of participants with baseline CAD scores for pulmonary TB above the manufacturer’s recommended thresholds (or in the case of CAD4TBv7, a commonly used threshold). Each participant with a score above recommended thresholds at which a diagnosis of TB is likely is represented as a human figure. Each figure is accurately represented as male or female, the proportions of participants with prevalent (routine and enhanced) and incident TB are shown and those with symptoms at baseline or a history of previous TB highlighted. Numbers represent proportions (95% CI). The chest radiograph illustrations show an example of the typical output from each of the CAD products. Abbreviations: CAD, computer-aided detection; CAD4TBv7/CAD4TB, CAD4TB version 7; F, female; Lunit v3/Lunit INSIGHT CXR, Lunit INSIGHT CXR version 3.1.4.111; M, male; qXR v3/qXR, qXR version 3.0.0; TB, tuberculosis.

Between 8% (39/483) and 18% (85/483) of participants had CAD scores above the recommended threshold and, of those, 7–13% were diagnosed as prevalent cases with routine sampling. However, 30–35% of those above the threshold and not diagnosed routinely were subsequently diagnosed and treated for TB either as enhanced sampling prevalent cases (54–69%) or incident cases (31–46%) (ie cannot be considered as a “false positive”). Of those diagnosed routinely, 67–80% were symptomatic at baseline in comparison to 14–36% of those diagnosed with enhanced sampling and 20–25% of those diagnosed with incident TB (*P* = .04 [qXRv3], .13 [CAD4TBv7], .18 [Lunitv3]) (Figure 5). For participants above the threshold not diagnosed or treated for TB over 5 years, all had a previous history of TB with CAD4TBv7 (25/25) and qXRv3 (22/22), and 87% (46/53) with Lunitv3 (Figures 4 and 5).

For each product, we calculated thresholds using the WHO target product profile (TPP) optimal sensitivity (0.95) and specificity (0.8) for a TB triage test for detecting any prevalent TB case. These derived thresholds were substantially lower than those recommended or currently used in practice (see Supplementary Table 4)

Of the 247 HIV-uninfected, asymptomatic participants in the biomarker subgroup, 244 used CRP, 247 used ESR, 240 used MTB-HR, and 245 used QuantiFERON-Gold. Overall, 18 of 247 participants were diagnosed and treated for TB (6 prevalent [1 routine, 5 enhanced] and 12 with incident TB). In this subgroup for the detection of asymptomatic prevalent TB, the AUC of the blood-based biomarkers was lower than for CAD software. For Lunitv3, CAD4TBv7, and qXRv3, respectively (n = 247), the AUCs were .98 (.93–1), .95 (.89–1), and .90 (.74–1). In comparison, the AUC for CRP was .75 (.55–.96) (*P* = .02, .03, and .14 [compared to Lunitv3, CAD4TBv7, and qXRv3, respectively]), for ESR was .78 (.60–.96) (*P* = .003, .007, and .003), for QFT-Gold was .58 (.39–.78) (*P* = .001, .004, and .07), and for MTB-HR was .67 (.39–.96) (*P* = .01, .01, and .06). Sensitivity analyses for 238 participants excluding 9 with at least 1 missing biomarker result did not show any differences in AUC for prevalent cases. With asymptomatic prevalent TB intensively screened out, diagnostic performance for incident TB was limited with no significant differences between the CAD software products (AUC, .64–.72) and blood biomarkers (AUC, .55–.61). Incorporating each blood biomarker individually into a predictive model with CAD software also did not significantly improve the AUC to detect prevalent or incident TB. In an exploratory analysis, blood testing in those with CAD above the threshold had limited impact on the likelihood ratio for TB (Supplementary Figure 2, Supplementary Tables 5 and 6).

## DISCUSSION

This is the first study reporting on the performance of CAD in which CXR, symptom screen, and routine and enhanced sputum investigation have been undertaken to screen all consenting HHCs for TB with subsequent follow-up for incident disease. We showed that routine confirmatory testing with single spontaneous Xpert MTB/RIF to diagnose pulmonary TB captures only 30% (7/23) of total prevalent disease. Chest X-ray screening with CAD interpretation had excellent diagnostic performance to detect all those with prevalent disease (AUC, .87–.91). Following baseline enhanced sputum investigation, the performance of CAD to identify incident cases was limited (AUC range, .60–.65). However, using common thresholds, we also showed that 30–35% of those not positive by routine sampling were either sputum positive by more intensive investigations or were diagnosed with incident TB over the follow-up period, with almost all of the remainder having previous TB. Furthermore, we highlighted that, despite excellent AUCs, the recommended CAD threshold sensitivity is relatively low (71–86% for routine prevalent and 57–87% for all prevalent) and that thresholds could be lowered. Finally, we showed in a subgroup of asymptomatic, HIV-uninfected participants that the diagnostic performance of all CAD products to detect asymptomatic prevalent TB was generally superior to a range of blood-based biomarkers and did not substantially improve with the addition of any of these blood-based biomarkers.

We have shown for the first time in a contemporary setting that those who have CXR changes suggestive of TB but who are bacteriologically negative by routine approaches have a similar risk of progressing to bacteriologically positive disease, as shown in the recently published historical systematic review [16]. Our findings also suggest that the potential of CAD-CXR– based screening is not being maximized. Computer-aided detection screening can identify a population with early, often asymptomatic, stages of TB who are not readily bacteriologically confirmed with a single sputum sample; although a proportion can be diagnosed with repeated/more intensive sputum sampling, others cannot and require follow-up and repeated testing. A number of blood tests proposed as potentially being diagnostically/prognostically useful in this setting do not appear to improve on the performance of baseline CXR in those without symptoms. These tests are markers of inflammation or interferon stimulation and may have improved performance in those with symptoms, which we were unable to assess.

Our results build on existing literature on CAD diagnostic performance for screening. A systematic review conducted by Harris and colleagues [13] in 2019 found 3 diagnostic accuracy studies that used a microbiological reference standard in screening settings [20–22]. However, the authors identified significant sources of bias and methodological limitations in these studies.

Our study has several strengths that differentiate it from other diagnostic accuracy studies. We included all HHCs who were eligible and who provided consent for TB screening and performed a thorough examination of sputum with multiple samples for investigation at baseline in all participants, and followed up participants over a 5-year period. This enabled us to avoid misclassification of TB cases; however, at the same time, we were able to compare with routine sampling to ensure wider applicability of our results. This was also the first diagnostic accuracy study to incorporate and compare a range of blood-based biomarkers, including the point-of-care Xpert MTB-HR test alongside assessment of CAD, although this analysis was restricted to a subset of HIV-uninfected participants without symptoms.

Our study has some limitations. Although we demonstrated that lower thresholds could be used and still meet WHO TPP, such cutoffs were not externally validated. We screened for symptoms of TB using unexplained cough for 2 or more weeks, fever, night sweats, and weight loss, for both HIV-infected and -uninfected persons rather than the recommended WHO four symptom screen (W4SS) in HIV infection [8]. This approach was taken for consistency and all participants were investigated at baseline regardless of symptoms, thus reducing the bias in our study findings. We used Xpert MTB/RIF as the confirmatory molecular test. Xpert Ultra has a lower limit of detection and may be more sensitive but at the expense of specificity. This study was not powered to assess the impact of incorporating additional biomarkers on diagnostic accuracy and needs further assessment. Our study was performed in HHCs in a high-burden setting where there is increased risk of reinfection; and although it is likely that many of our findings would be applicable in other screening settings, additional studies in low-burden settings should be undertaken.

Following the updated WHO screening guidelines we will continue to see scale-up of CAD-CXR screening. Our work has shown that, although CAD software is diagnostically accurate, its utility is not being maximized as approximately 30% of those with CAD scores above the threshold with negative Xpert MTB/RIF on a spontaneous sputum sample either have prevalent TB diagnosed by intensive sampling or develop incident TB over time. Further work is needed to establish how best to follow up and manage these patients.

## Supplementary Data

Supplementary Materials are available at *Clinical Infectious Diseases* online. Consisting of data provided by the authors to benefit the reader, the posted materials are not copyedited and are the sole responsibility of the authors, so questions or comments should be addressed to the corresponding author.

## Supplementary Material

ciae528_Supplementary_Data

## Notes

***Author Contributions.*** The study was conceived by H. E., S. V. K., M. R., A. K. C., and R. J. W. The study methods were determined by H. E., L. M., S. V. K., M. Q., and A. K. C. Clinical activities and data collection were undertaken by R. D., R. C., A. T., K. S., R. T. G., N. O. D., A. J., E. D., B. S., S. M., and F. T. Laboratory activities were undertaken by F. L., M. J., N. Y., S. G., S. A., and T. S. The data were curated by L. M., E. D., A. K. C., and A. J. Analysis was performed by L. M. with supervision and support from H. E., S. V. K., and M. Q. The first draft of the manuscript was prepared by L. M., H. E., and S. V. K., with all co-authors subsequently reviewing and contributing to the manuscript.

***Financial support.*** This work was supported by a Medical Research Council award (grant number: MR/V00476X/1) to H. E. H. E. is partially supported by a Medical Research Council unit grant (grant number: MC UU 00004/04). R. J. W. is supported via the Francis Crick Institute, which receives funding from Cancer Research UK (grant number: FC2112), the Medical Research Council (grant number: CC2112), and Wellcome Trust (grant number: CC2112). He also receives funding from Wellcome (grant number: 203135), South African Medical Research Council, and the Bill and Melinda Gates Foundation. Work from authors affiliated with FIND (M. R., S. V. K.) was supported with funding provided by the Bundesministerium für Bildung und Forschung **(**German Fedral Ministry for Education and Research) through KfW Development Bank. For the purposes of open access, the authors have applied a CC-BY public copyright to any author-accepted manuscript arising from this submission.
