# Supplementary material for: Diagnostic Accuracy of Chest X-ray Computer-Aided Detection Software for Detection of Prevalent and Incident Tuberculosis in Household Contacts
Source: Clin Infect Dis. 2024 Dec 18;80(3):626–36. doi: 10.1093/cid/ciae528 (PMC11912973; doi:10.1093/cid/ciae528)
Supplement: ciae528_Supplementary_Data [file ciae528_supplementary_data.docx]

**Supplementary data**

Diagnostic accuracy of Chest X-Ray Computer Aided Detection software for detection of prevalent and incident tuberculosis in household contacts

Liana Macpherson, Sandra V. Kik, Matteo Quartagno, Francisco Lakay, Marche Jaftha, Nombuso Yende, Shireen Galant, Saalikha Aziz^3^, Remi Daroowala, Richard Court, Arshad Taliep, Keboile Serole, Rene T. Goliath, Nashreen Omar Davies, Amanda Jackson, Emily Douglass, Bianca Sossen, Sandra Mukasa, Friedrich Thienemann, Taeksun Song, Morten Ruhwald, Robert J. Wilkinson, Anna K. Coussens, Hanif Esmail on behalf of the Imaging of TB household contacts group.

Table of Contents

[Members of the Imaging of TB household contacts group 2](#_Toc182817831)

[Details of household contact recruitment 2](#_Toc182817832)

[Inclusion and exclusion criteria – n=250 biomarker subgroup: 3](#_Toc182817833)

[Xpert MTB-HR Methodology 3](#_Toc182817834)

[Additional CAD methodology 6](#_Toc182817835)

[Supplementary Table 1: Microbiology results for participants with prevalent TB (n=23) 7](#_Toc182817836)

[Supplementary Figure 1: AUC ROCs for incident TB over time for each CAD software. 8](#_Toc182817837)

[Supplementary Table 2: Subgroup analysis - AUC ROC for participants with and without HIV infection and for those with and without a previous history of TB. 9](#_Toc182817838)

[Supplementary Table 3: The sensitivity and specificity of each CAD software using the manufacturer recommended (or commonly used) threshold. 10](#_Toc182817839)

[Supplementary Table 4: Thresholds derived from the WHO target product profile optimal sensitivity (0.95) and specificity (0.8) for a TB triage test. 11](#_Toc182817840)

[Supplementary Table 5: AUC ROC for each CAD software for detecting prevalent and incident TB, in combination with biomarkers: 12](#_Toc182817841)

[Supplementary Figure 2: CAD score and biomarkers (host response blood test, CRP, ESR and QuantiFERON result for 247 HIV uninfected, asymptomatic participants for each CAD software. 13](#_Toc182817842)

[Supplementary Table 6: 2-step diagnostic strategy – Blood testing in those with testing CAD score > threshold 17](#_Toc182817843)

## Members of the Imaging of TB household contacts group

| First name and Inital | Surname | Affiliation |
| --- | --- | --- |
| Clifton E. | Barry III | Tuberculosis Research Section, Laboratory of Clinical Immunology and Microbiology, National Institute of Allergy and Infectious Diseases, National Institutes of Health, Bethesda, MD. |
| Jerrold J. | Ellner | Rutgers- New Jersey Medical School, Center for Emerging Pathogens, Newark, NJ, United States. |
| JoAnne L. | Flynn | Department of Microbiology and Molecular Genetics, University of Pittsburgh School of Medicine, PA 15261, United States of America |
| Torben | Heinsohn | Department of Epidemiology, Helmholtz Centre for Infection Research, Braunschweig, Germany. |
| C. Robert | Horsburgh Jr. | Department of Medicine, Boston University School of Medicine, Boston, MA, USA; Departments of Epidemiology, Biostatistics and Global Health, Boston University School of Public Health, Boston, MA, USA |
| Karen R. | Jacobson | Department of Medicine, Boston University School of Medicine, Boston, MA, USA; Departments of Epidemiology, Biostatistics and Global Health, Boston University School of Public Health, Boston, MA, USA |
| Stephanus T. | Malherbe | Department of Science and Technology/National Research Foundation Centre of Excellence in Biomedical Tuberculosis Research, South African Medical Research Council for Tuberculosis Research, Division of Molecular Biology and Human Genetics, Department of Biomedical Sciences, Faculty of Health Sciences, Stellenbosch University, Cape Town, South Africa. |
| Padmini | Salgame | Rutgers- New Jersey Medical School, Center for Emerging Pathogens, Newark, NJ, United States. |
| Dylan | Sheerin | The Walter and Eliza Hall Institute of Medical Research, Parkville, Victoria 3052, Australia Department of Medical Biology, University of Melbourne, Parkville, 3052, Australia. |
| Elizabeth | Streicher | Department of Science and Technology/National Research Foundation Centre of Excellence in Biomedical Tuberculosis Research, South African Medical Research Council for Tuberculosis Research, Division of Molecular Biology and Human Genetics, Department of Biomedical Sciences, Faculty of Health Sciences, Stellenbosch University, Cape Town, South Africa. |
| Mpho | Tlala | Department of Science and Technology/National Research Foundation Centre of Excellence in Biomedical Tuberculosis Research, South African Medical Research Council for Tuberculosis Research, Division of Molecular Biology and Human Genetics, Department of Biomedical Sciences, Faculty of Health Sciences, Stellenbosch University, Cape Town, South Africa. |
| Laura E. | Via | Tuberculosis Research Section, Laboratory of Clinical Immunology and Microbiology, National Institute of Allergy and Infectious Diseases, National Institutes of Health, Bethesda, MD Tuberculosis Imaging Program, Division of Intramural Research, National Institute of Allergy and Infectious Diseases, National Institutes of Health, Bethesda, MD. |
| Gerhard | Walzl | Department of Science and Technology/National Research Foundation Centre of Excellence in Biomedical Tuberculosis Research, South African Medical Research Council for Tuberculosis Research, Division of Molecular Biology and Human Genetics, Department of Biomedical Sciences, Faculty of Health Sciences, Stellenbosch University, Cape Town, South Africa. |
| Robin | Warren | Department of Science and Technology/National Research Foundation Centre of Excellence in Biomedical Tuberculosis Research, South African Medical Research Council for Tuberculosis Research, Division of Molecular Biology and Human Genetics, Department of Biomedical Sciences, Faculty of Health Sciences, Stellenbosch University, Cape Town, South Africa. |
| James | Warwick | Division of Nuclear Medicine, Department of Medical Imaging and Clinical Oncology, Stellenbosch University, Cape Town, South Africa |

## Details of household contact recruitment

Index cases aged ≥15 years with at least rifampicin resistant pulmonary tuberculosis (TB) confirmed by Xpert MTB/RIF or culture consented to a household visit and to contact their household members and close contacts. Household contacts (HHC) were defined as individuals sleeping in the same dwelling or room and/or providing themselves jointly with food or other essentials for living during the day with the index case for at least 7 days during the 3 months prior to the index case being diagnosed with TB. All HHC ≥18 years were offered an appointment at the study clinic for TB screening. Written, informed consent was obtained from all index cases and HHC participants.

## Inclusion and exclusion criteria – n=250 biomarker subgroup:

The biomarker subgroup was part of a wider substudy exploring the radiological and immunological characteristics of subclinical TB and also included PET/CT imaging and additional sampling. Other findings from this work will be reported separately. The inclusion/exclusion criteria were as follows:

*Inclusion Criteria*

1. Household contact of DR-TB index case
2. Age 18 years or over
3. Consents to participating in study
4. Willing to undergo HIV counselling and testing (HCT)

*Exclusion Criteria*

1. HIV infection
2. On TB treatment at the time of screening
3. Symptoms or signs of active TB
4. Symptoms or signs of acute illness
5. Age >65 years
6. Smoker >30 pack years
7. Diagnosis of malignancy
8. Diagnosis of chronic lung infection other than TB (e.g., non-tuberculosis mycobacteria [NTM], Fungal)
9. Diagnosis of chronic inflammatory condition associated with pulmonary pathology (e.g., Sarcoidosis, Rheumatoid Arthritis, Wegener’s granulomatosis, bronchiectasis)
10. Inhaled or systemic steroid use within previous 2 weeks
11. Breast feeding, pregnant, or planning pregnancy over next 3 months
12. Unable to be followed up for 6 months
13. Uncontrolled diabetes mellitus

## Xpert MTB-HR Methodology

***Xpert MTB-HR Tempus Tube validation cohort***

The Xpert Mtb-HR cartridge is designed to be used with 100 μl of capillary finger prick blood or pelleted Paxgene blood cells resuspended in TB HR Lysis Buffer added directly to the cartridge. To validate its use with stored Tempus tube blood a comparison was performed between results obtained using fresh capillary blood and stored Tempus tubes taken from 49 individuals. Participants included 19 individuals newly diagnosed with TB at the Site B Ubuntu clinic in Khayelitsha (6 HIV-infected, 13 HIV uninfected) and 30 healthy controls, confirmed not to have TB recruited via the Site B HIV wellness clinic, including 12 HIV infected individuals previously established on ART and 18 individuals confirmed HIV uninfected following HIV counselling and testing, performed by the wellness clinic staff. Written informed consent was obtained from all participants following human research ethical approval received from University of Cape Town Faculty of Health Sciences (449/2014) and University College London (19219/001).

***Xpert MTB-HR Tempus Tube vs capillary blood comparison***

Finger prick blood was taken using a Minivette (BD) collecting 100 μl of whole blood that was added directly to the Xpert MTB-HR cartridge within 15 min of blood draw. Following the fingerpick, venus blood was also collected in a Tempus Tube that was stored at -80^o^C (for > 7 days until processed) and an EDTA tube that was stored at RT on the day until the capillary MTB-HR result was confirmed valid. If an invalid result was recorded the test was repeated adding 100 μl of EDTA whole blood directly to a new cartridge. To run the Tempus tubes, following overnight thawing at 4^o^C, tubes were inverted 2-3 times and 380 μl of Tempus blood transferred to 1.5 ml Eppendorf and centrifuged at 3000g for 5 min. Supernatant was removed, cell pellet resuspended in 100 μl of TB HR Lysis Buffer (Cepheid). Samples were vortexed for 10 sec to dissolve the pellet and then 100 μl transferred to the Xpert MTB-HR cartridge. All cartridges were run within 30 min of blood addition on a GeneXpert instrument using the TB Host Response Alpha software module. Results indicated no significant difference in fresh blood vs stored Tempus blood results for the HIV-infected and HIV-uninfected controls and only a small decrease in median LDA (fresh 0.48 vs Tempus 0.30, p=0.029) for the TB group (A below). Based on the LDA threshold cutoff of <2 indicating TB, 16/19 (84%) TB patients had LDA <2 for fresh blood and 17/19 (89%) for Tempus blood. All stored Tempus tubes from HHC in the biomarker substudy were therefore run as detailed above.

***Xpert MTB-HR Tempus Tube vs extracted RNA comparison***

Of the 247 HHC in the biomarker substudy that had 2 Tempus tubes stored, 44 had already had RNA extracted from both Tempus tubes and had no Tempus tubes remaining. To validate equivalent results using extracted RNA from the same Tempus tube, one Tempus tube from each of 9 individuals with 2 remaining tubes were thawed and first the Tempus tube blood analysed on the Xpert MTB-HR cartridge, then RNA extracted from the same Tempus tube using the Norgen Preserved Blood RNA kits (for Tempus Tubes), following manufacturers protocol including DNAse treatment (Norgen). Three volumes of RNA to be added to the cartridge were then tested resuspending to a total of 100 μl using TB HR Lysis Buffer: 2 μl, 1,8 μl and 0.5 μl. Results indicated a small significant higher LDA value using 2 μl and no difference using 0.5 μl (B below). Given the increased potential of pipetting error when adding 0.5 μl, 1 μl + 99 μl TB HR Lysis Buffer was used to run the 44 samples with only RNA remaining.

**Validation of using Tempus tube blood and extracted RNA in the Xpert MTB-HR cartridge**. (**A**) LDA results from asymptomatic HIV-uninfected (n=18), asymptomatic HIV-infected (n=12) no TB controls and symptomatic newly diagnosed TB patients (n=19), comparing results between fresh blood and stored Tempus tube blood. Median indicated by line, analysed by Wilcoxon matched-pairs test. (**B**) LDA results from asymptomatic HIV-uninfected (n=9) person comparing using Tempus tube blood and different volumes of RNA extracted from the same Tempus tube. Friedman test with Dunn's multiple comparison testing.

## Additional CAD methodology

CAD software was installed by the companies on local servers (one for each software) that are under the control of FIND, one of the partners in this study. After successful installation, access to the servers was lifted for the CAD vendors to allow for an independent evaluation. Anonymised digital images were uploaded via a secure server to FIND and then processed by the CAD software. No images were shared with the software manufacturers. The CAD output consists of a score along a continuous scale (CAD4TB and Lunit INSIGHT CXR 0-100, qXR 0-1), where high scores indicate a higher probability of active TB.

Both qXRv3 and Lunit INSIGHT CXRv3 come with manufacturer recommended threshold scores for TB (0.5 and 15 respectively), above which the X-ray is considered likely compatible with TB and below which it is not. For CAD4TBv7 developer recommend that the threshold is optimised for for setting and program needs with lower scores used if wanting to optimise sensitivity and higher scores if wanting to optimise specificity.^[[1]](#footnote-2)^ In practice, this cut off ranges between ~50-70 have been used and we used the lower score of 50 for this analysis.

## Supplementary Table 1: Microbiology results for participants with prevalent TB (n=23)

| **Smear status:**  Scanty  1+  2+  3+ | 10/23 (43%) smear positive  2 (9%)  2 (9%)  5 (22%)  1 (4%) |
| --- | --- |
| *Mtb* detected by Xpert MTB/RIF in at least 1 baseline sample | 14/23 (61%) Xpert positive |
| Proportion with any culture positive sample | 20/23 (87%) culture positive |

Table shows the smear, Xpert MTB/RIF and culture status for 23 participants with prevalent TB (routine or enhanced).

## Supplementary Figure 1: AUC ROCs for incident TB over time for each CAD software.

CAD4TBv7

qXRv3

Lunit INSIGHT CXRv3


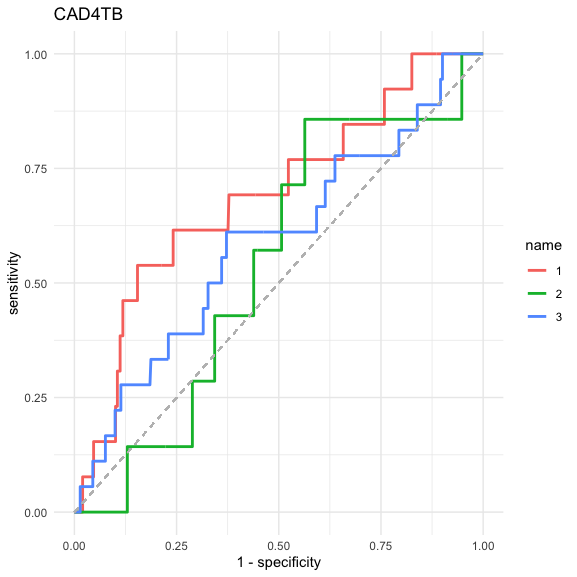

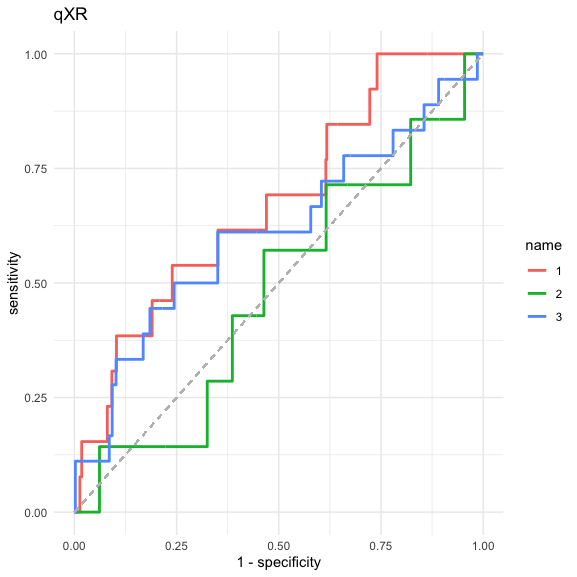

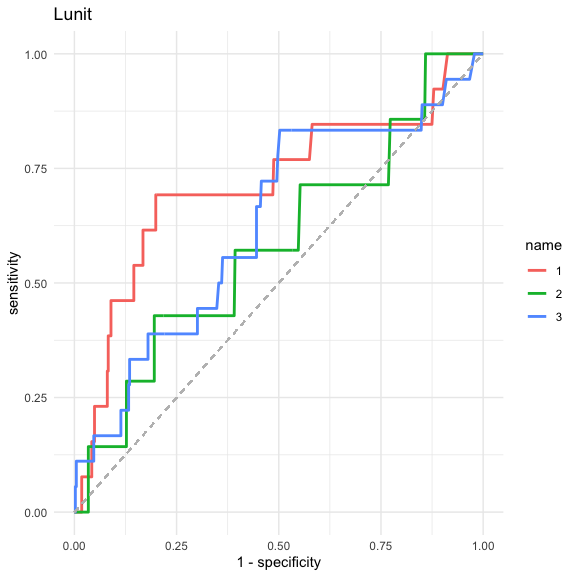


>0 - ≤12 months

>12 - ≤24 months

>24 months

|  | AUC ROC for CAD4TB (95% CI) | AUC ROC for qXR (95% CI) | AUC ROC for Lunit INSIGHT CXR (95% CI) |
| --- | --- | --- | --- |
| Incident TB from 1-12 months **(n= 460)** | 0.69 (0.53-0.85) | 0.67 (0.52-0.82) | 0.71 (0.54-0.89) |
| Incident TB from 13-24 months **(n= 447)** | 0.543 (0.35-0.74) | 0.48 (0.25-0.71) | 0.58 (0.34-0.82) |
| Incident TB from 25 months **(n= 440)** | 0.61 (0.45-0.77) | 0.58 (0.44-0.73) | 0.63 (0.49-0.77) |

Figure show the performance of each of the 3 CAD software for incident TB by timing of diagnosis; >0 - ≤12 months, <12 - ≤24 months and >24 months.

## Supplementary Table 2: Subgroup analysis - AUC ROC for participants with and without HIV infection and for those with and without a previous history of TB.

|  | **Previous TB (n=109)** | **No previous TB (n=374)** | ***P value*** |
| --- | --- | --- | --- |
| **AUC for all prevalent TB**  CAD4TBv7  qXRv3  Lunit INSIGHT CXRv3 | 0.65 (0.43-0.87)  0.69 (0.54-0.85)  0.70 (0.57-0.83) | 0.94 (0.84-1)  0.90 (0.77-1)  0.93 (0.82-1) | *0.02*  *0.04*  *0.01* |
| **AUC for all incident TB**  CAD4TBv7  qXRv3  Lunit INSIGHT CXRv3 | 0.50 (0.31-0.69)  0.49 (0.28-0.71)  0.54 (0.33-0.74) | 0.62 (0.50-0.74)  0.65 (0.52-0.77)  0.69 (0.56-0.81) | *0.31*  *0.21*  *0.23* |

|  | **HIV infected (n=136)** | **HIV uninfected (n=340)** | ***P value*** |
| --- | --- | --- | --- |
| **AUC for all prevalent TB**  CAD4TBv7  qXRv3  Lunit INSIGHT CXRv3 | 0.78 (0.59-0.96)  0.85 (0.68-1)  0.85 (0.69-1) | 0.94 (0.90-0.98)  0.91 (0.82-0.997)  0.96 (0.93-0.99) | *0.09*  *0.5*  *0.18* |
| **AUC for all incident TB**  CAD4TBv7  qXRv3  Lunit INSIGHT CXRv3 | 0.63 (0.51-0.76)  0.63 (0.50-0.76)  0.64 (0.49-0.79) | 0.57 (0.41-0.73)  0.64 (0.48-0.79)  0.66 (0.52-0.80) | *0.55*  *0.95*  *0.84* |

Table shows the comparison of diagnostic performance for prevalent and incident TB in those with and without a previous TB history (top) and with and without HIV history (bottom) for the 3 different CAD softwares.

## Supplementary Table 3: The sensitivity and specificity of each CAD software using the manufacturer recommended (or commonly used) threshold.

|  | CAD4TBv7 | | | qXRv3 | | | Lunit INSIGHT CXRv3 | | |
| --- | --- | --- | --- | --- | --- | --- | --- | --- | --- |
|  | Routine prevalent TB cases | All prevalent TB cases | All TB cases | Routine prevalent TB cases | All prevalent TB cases | All TB cases | Routine prevalent TB cases | All prevalent TB cases | All TB cases |
| Threshold | **50** | | | **0.5** | | | **15** | | |
| Sensitivity | 0.71 (0.29-0.96) | 0.70 (0.47-0.87) | 0.34 (0.23-0.48) | 0.71 (0.29-0.96) | 0.57 (0.34-0.77) | 0.52 (0.39-0.65) | 0.86 (0.42-0.996) | 0.87 (0.66-0.97) | 0.52 (0.39-0.65) |
| Specificity | 0.91 (0.89-0.94) | 0.93 (0.91-0.96) | 0.94 (0.91-0.96) | 0.93 (0.90-0.95) | 0.94 (0.92-0.96) | 0.98 (0.97-0.99) | 0.83 (0.80-0.87) | 0.86 (0.82-0.89) | 0.87 (0.84-0.90) |
| PPV | 0.11 (0.07-0.17) | 0.35 (0.26-0.45) | 0.46 (0.33-0.58) | 0.13 (0.08-0.21) | 0.33 (0.23-0.46) | 0.82 (0.68-0.91) | 0.07 (0.05-0.10) | 0.24 (0.19-0.29) | 0.38 (0.30-0.46) |
| NPV | 0.995 (0.99-0.999) | 0.98 (0.97-0.99) | 0.91 (0.89-0.92) | 0.995 (0.986-0.999) | 0.98 (0.96-0.99) | 0.93 (0.92-0.95) | 0.998 (0.98-0.999) | 0.99 (0.98-0.997) | 0.93 (0.90-0.94) |
| TP | 5 | 16 | 21 | 5 | 13 | 32 | 6 | 20 | 32 |
| FP | 41 | 30 | 25 | 35 | 27 | 70 | 79 | 65 | 53 |
| TN | 435 | 430 | 397 | 441 | 433 | 352 | 397 | 395 | 369 |
| FN | 2 | 7 | 40 | 2 | 10 | 29 | 1 | 3 | 29 |

PPV = positive predictive value, NPV = negative predictive value

TP = True positive, FP = False positive, TN = True negative, FN = False negative.

Table shows the diagnostic performance of the recommended or commonly used thresholds for CAD4TBv7 (≥50), qXRv3 (≥0.50) and Lunitv3 (≥15)

## Supplementary Table 4: Thresholds derived from the WHO target product profile optimal sensitivity (0.95) and specificity (0.8) for a TB triage test.

|  | CAD4TBv7 | | qXRv3 | | Lunit INSIGHT CXRv3 | |
| --- | --- | --- | --- | --- | --- | --- |
|  | All prevalent TB cases | | All prevalent TB cases | | All prevalent TB cases | |
| **Threshold** | **3.36** | **25.6** | **0.0097** | **0.038** | **5.11** | **4.47** |
| Sensitivity | 0.95 (fixed) | 0.83 | 0.95 (fixed) | 0.87 | 0.95 (fixed) | 0.96 |
| Specificity | 0.22 | 0.8 (fixed) | 0.46 | 0.8 (fixed) | 0.81 | 0.8 (fixed) |
| PPV | 0.06 | 0.17 | 0.08 | 0.18 | 0.20 | 0.19 |
| NPV | 0.99 | 0.99 | 0.995 | 0.99 | 0.997 | 0.997 |
| TP | 22 | 19 | 22 | 20 | 22 | 22 |
| FP  Incident cases | 361  32 (8.9%) | 91  14 (15.3%) | 250  24 (9.6%) | 92  15 (16.3%) | 87  16 (18.4%) | 92  16 (17.4%) |
| TN | 99 | 369 | 210 | 368 | 373 | 368 |
| FN | 1 | 4 | 1 | 3 | 1 | 1 |

PPV = positive predictive value, NPV = negative predictive value

TP = True positive, FP = False positive, TN = True negative, FN = False negative.

Incident cases = number (percentage) of cases considered false positive that subsequently develop incident TB over follow-up

Table shows diagnostic performance of thresholds optimised for either sensitivity or specificity for 3 CAD software. Using a sensitivity of 0.95 the derived thresholds were 3.36 (specificity 0.22), 0.0097 (specificity 0.46), and 5.11 (specificity 0.81) for CAD4TBv7, qXRv3 and Lunit INSIGHT CXRv3 respectively. Using a specificity of 0.8 the derived thresholds were 25.6 (sensitivity 0.83), 0.038 (sensitivity 0.87), 4.47 (sensitivity 0.96) for CAD4TBv7, qXRv3 and Lunit INSIGHT CXRv3 respectively. These data suggest that current thresholds used by manufacturers could potentially be lowered in a screening setting where more intensive sputum sampling approaches are taken.

## Supplementary Table 5: AUC ROC for each CAD software for detecting prevalent and incident TB, in combination with biomarkers:

|  | **All prevalent TB cases** | **Incident TB cases** |
| --- | --- | --- |
| CRP (n=245) | 0.75 (0.55-0.96) | 0.59 (0.45-0.73) |
| ESR (n=249) | 0.78 (0.60-0.96) | 0.61 (0.46-0.76) |
| QuantiFERON (n=247) | 0.58 (0.39-0.78) | 0.55 (0.38-0.72) |
| Host response (n=242) | 0.67 (0.39-0.96) | 0.59 (0.40-0.79) |
| CAD4TBv7  CAD4TB + CRP  CAD4TB + ESR  CAD4TB + QuantiFERON  CAD4TB + HR | 0.95 (0.89-1)  0.94 (0.87-1)  0.94 (0.83-1)  0.98 (0.95-0.999)  0.93 (0.83-1) | 0.64 (0.42-0.85)  0.66 (0.43-0.89)  0.73 (0.58-0.89)  0.70 (0.50-0.90)  0.77 (0.62-0.92) |
| qXRv3  qXR + CRP  qXr + ESR  qXR + QuantiFERON  qXR + HR | 0.90 (0.74-1)  0.90 (0.73-1)  0.88 (0.65-1)  0.98 (0.95-0.998)  0.86 (0.59-1) | 0.68 (0.47-0.89)  0.71 (0.50-0.93)  0.77 (0.65-0.90)  0.71 (0.50-0.92)  0.82 (0.70-0.93) |
| Lunit INSIGHT CXRv3  Lunit INSIGHT CXR + CRP  Lunit INSIGHT CXR + ESR  Lunit INSIGHT CXR + QuantiFERON  Lunit INSIGHT CXR + HR | 0.98 (0.93-1)  0.93 (0.80-1)  0.90 (0.70-1)  0.995 (0.99-1)  0.89 (0.67-1) | 0.72 (0.54-0.91)  0.78 (0.61-0.95)  0.78 (0.65-0.90)  0.71 (0.52-0.91)  0.82 (0.71-0.93) |

Table showing diagnostic performance by AUC for CRP, ESR, QuantiFERON alone and in a model combining with each of 3 CAD software for prevalent and incident TB in asymptomatic, HIV uninfected household contacts.

## Supplementary Figure 2: CAD score and biomarkers (host response blood test, CRP, ESR and QuantiFERON result for 247 HIV uninfected, asymptomatic participants for each CAD software.

Host response blood test (n=240):

***Host-response score by CAD score for each CAD software:*** *Each dot represents a study participant. The horizontal blue line represents the threshold* ***below*** *which the host-response test is positive, the vertical blue line represents the manufacturer recommended threshold (or in the case of CAD4TB commonly used in the field) above which is consistent with radiographic TB.*


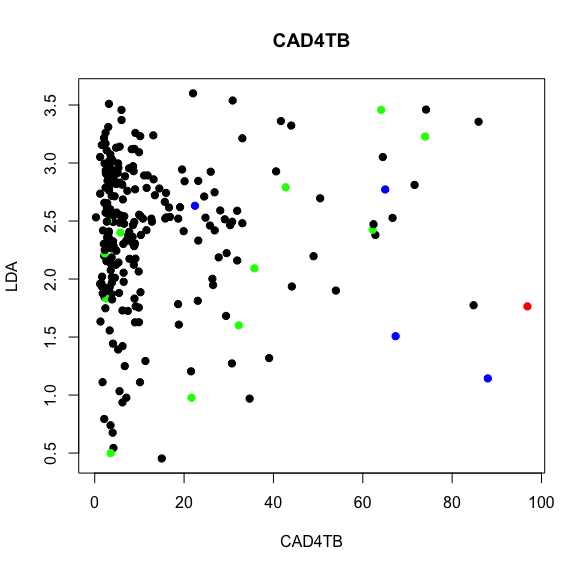

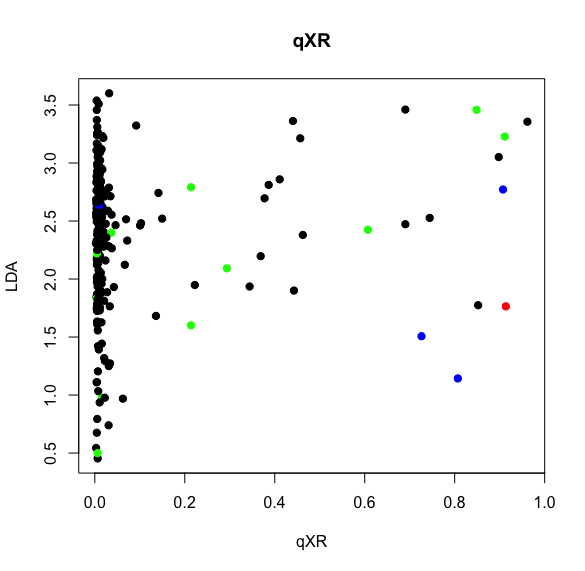

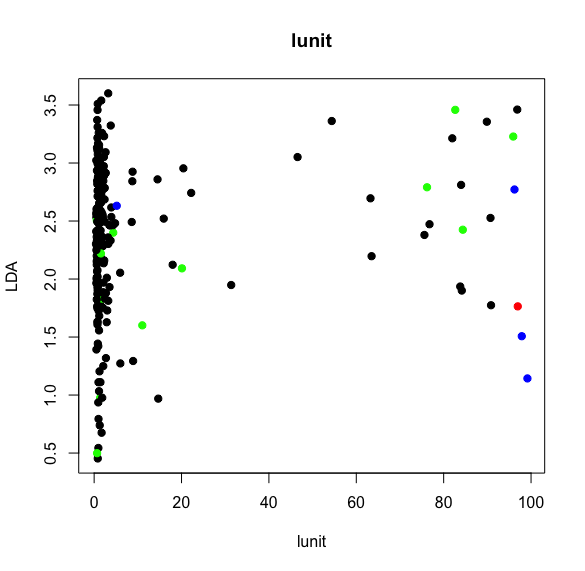


***= Routine prevalent TB case***

***= Enhanced prevalent TB case***

***= Incident TB case***

***= no TB***



Lunit INSIGHT CXR

CRP (n=244):


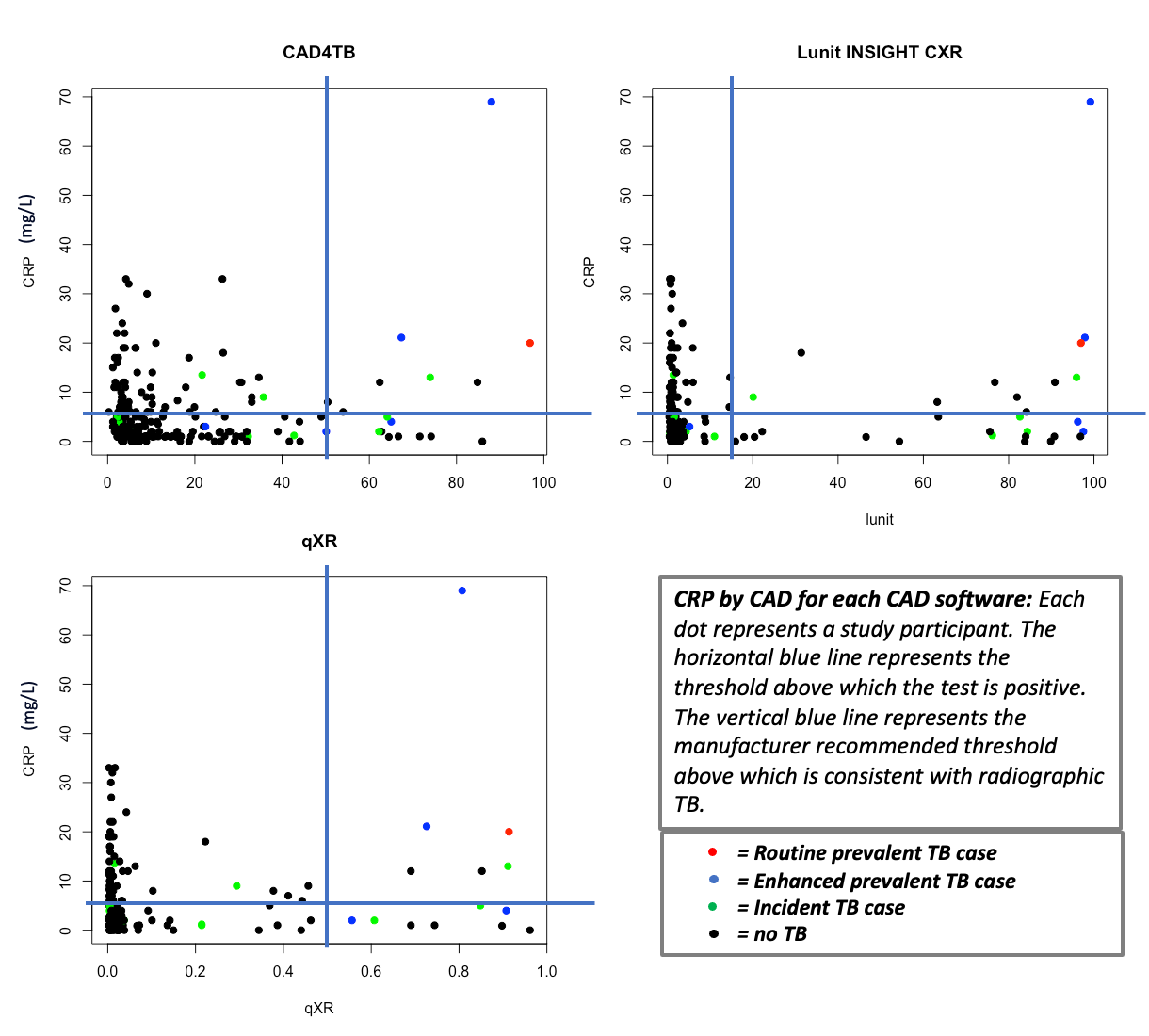


ESR (n=247):


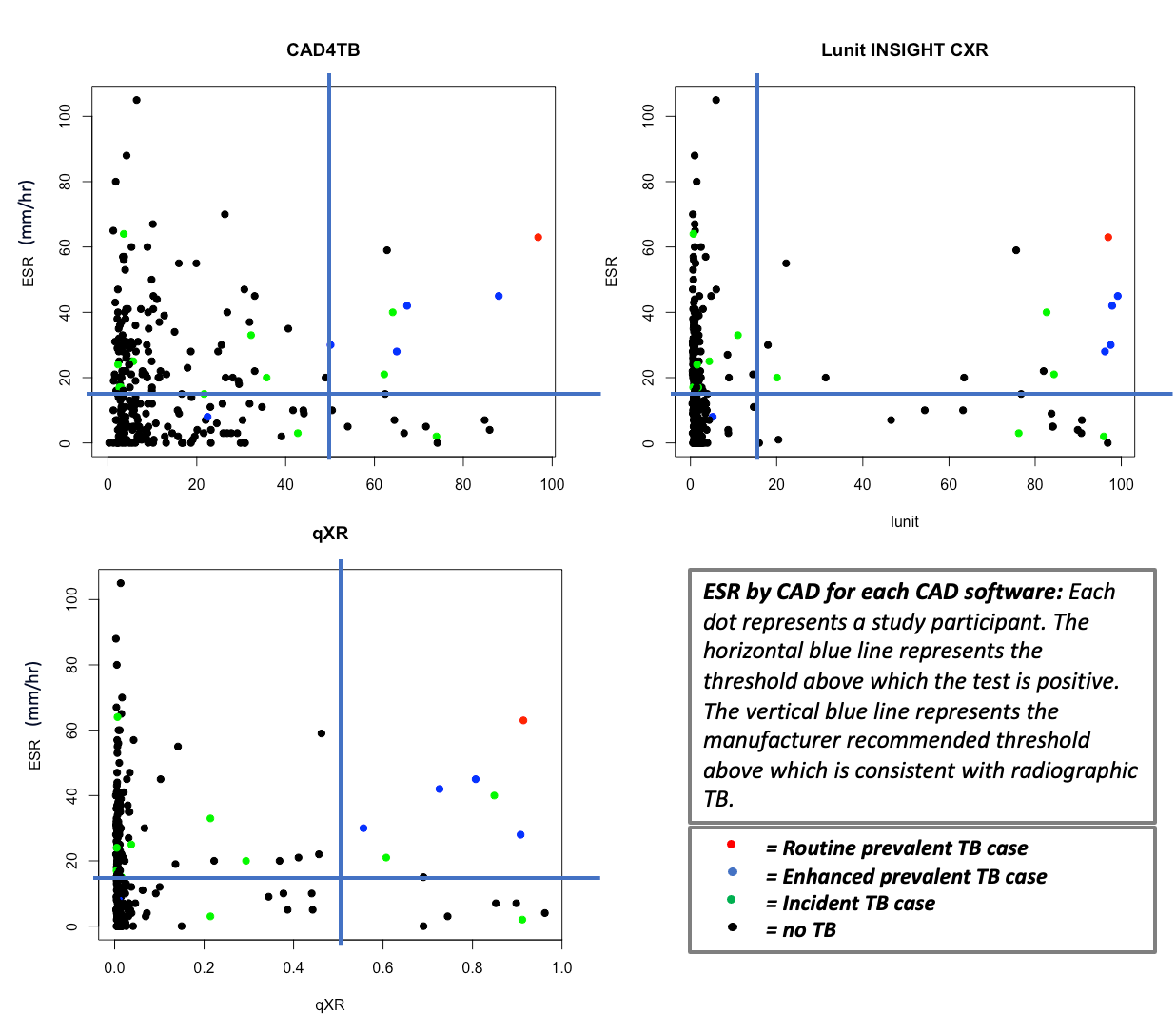


QuantiFERON (n=245):


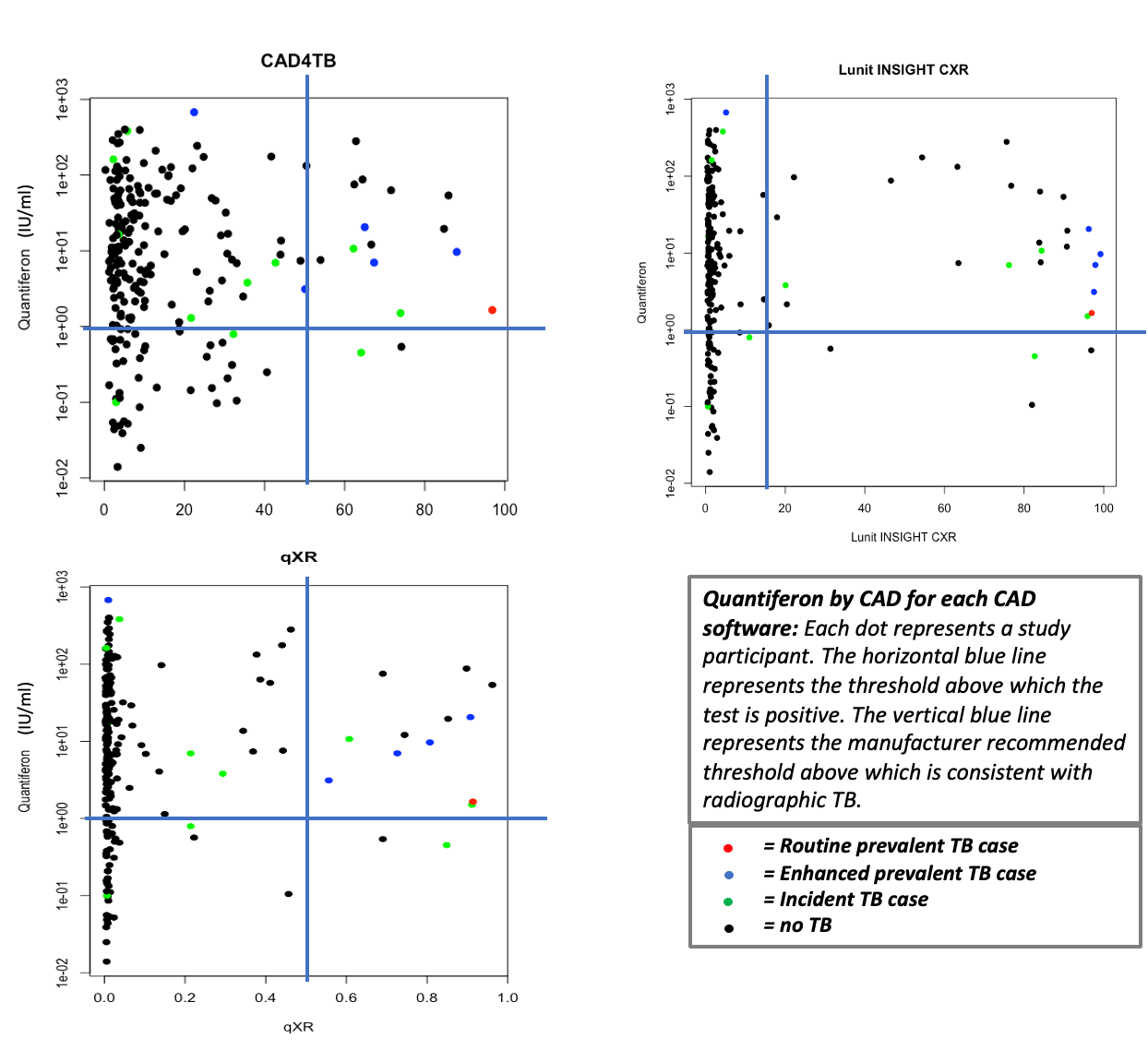


## Supplementary Table 6: 2-step diagnostic strategy – Blood testing in those with testing CAD score > threshold

We conducted an exploratory analysis (limited by small numbers and recognising participants were all asymptomatic) of a 2-step diagnostic strategy to evaluate if blood testing in those with CAD>threshold could better identify those with any TB (prevalent or incident) considering a positive result as CRP≥5mg/L (reflecting > normal range), ESR≥15mm/Hr (reflecting > normal range), QuantiFERON-Gold≥1.00IU/mL (a higher cut-off as essentially all participant >0.35IU/ml manufacturers cut-off) and Host Response blood test (MTB-HR) LDA≤2 (manufacturer suggested). Performance was encouraging for ESR≥15mm/Hr with sensitivity 80-87.5%, specificity 63.2-83.8% and a Likelihood Ratio for TB following a positive test result (LR+) 2.17-5.25. LR+ for CRP≥5mg/L was 1.56-1.88, QFN-Gold≥1IU/mL was 0.97-1.07 and MTB-HR LDA≤2 was 1.58-2.57 (see also Supplementary Figure 2).

Diagnostic performance of MTB-HR LDA ≤ 2 for any TB in those with CAD > threshold

| CAD4TBv7 ≥ 50  (n=17) | Sensitivity | 42.9% (9.9% -81.6%) |
| --- | --- | --- |
|  | Specificity | 80.0% (44.4%-97.5%) |
|  | Likelihood Ratio if positive | 2.14 (0.48 -9.66) |
| qXRv3 ≥ 0.5  (n=13) | Sensitivity | 42.9% (9.9%-81.6%) |
|  | Specificity | 83.3% (35.9% -99.6%) |
|  | Likelihood Ratio if positive | 2.57 (0.35-18.68) |
| Lunitv3≥ 15  (n=28) | Sensitivity | 33.3% (7.5% -70.1%) |
|  | Specificity | 78.9% (54.4%-93.9%) |
|  | Likelihood Ratio if positive | 1.58 (0.44-5.64) |

Diagnostic performance of CRP ≥ 5mg/L for any TB in those with CAD > threshold

| CAD4TBv7 ≥ 50  (n=18) | Sensitivity | 62.5% (24.5%-91.5%) |
| --- | --- | --- |
|  | Specificity | 60.0% (26.2% 87.8% |
|  | Likelihood Ratio if positive | 1.56 (0.62-3.96) |
| qXRv3 ≥ 0.5  (n=14) | Sensitivity | 62.5% (24.5%-91.5%) |
|  | Specificity | 66.7% (22.3%-95.7%) |
|  | Likelihood Ratio if positive | 1.88 (0.54 -6.56) |
| Lunitv3≥ 15  (n=29) | Sensitivity | 60.0% (26.2%-87.8%) |
|  | Specificity | 63.2% (38.4%-83.7%) |
|  | Likelihood Ratio if positive | 1.63 (0.75-3.54) |

Diagnostic performance of ESR ≥ 15mm/Hr for any TB in those with CAD > threshold

| CAD4TBv7 ≥ 50  (n=18) | Sensitivity | 87.5% (47.3%-99.7%) |
| --- | --- | --- |
|  | Specificity | 80.0% (44.4%-97.5% |
|  | Likelihood Ratio if positive | 4.38 (1.23-15.53) |
| qXRv3 ≥ 0.5  (n=14) | Sensitivity | 87.5% (47.3%-99.7%) |
|  | Specificity | 83.3% (35.9%-99.6%0 |
|  | Likelihood Ratio if positive | 5.25 (0.86-32.02) |
| Lunitv3≥ 15  (n=29) | Sensitivity | 80.0% (44.4%-97.5%) |
|  | Specificity | 63.2% (38.4%-83.7%) |
|  | Likelihood Ratio if positive | 2.17 (1.12-4.22) |

Diagnostic performance of QuantiFERON ≥ 1.0IU/mL for any TB in those with CAD > threshold

| CAD4TBv7 ≥ 50  (n=18) | Sensitivity | 87.5% (47.3%-99.7%) |
| --- | --- | --- |
|  | Specificity | 10.0% (0.3-44.5) |
|  | Likelihood Ratio if positive | 0.97 (0.7-1.36) |
| qXRv3 ≥ 0.5  (n=14) | Sensitivity | 87.5% (47.3%-99.7%) |
|  | Specificity | 16.7% (0.4%-64.1%) |
|  | Likelihood Ratio if positive | 1.05 (0.67-1.64) |
| Lunitv3≥ 15  (n=29) | Sensitivity | 90.0% (55.5%-99.7%) |
|  | Specificity | 15.8% (3.4%-39.6%) |
|  | Likelihood Ratio if positive | 1.07 (0.80-1.42) |

1. See Choosing the appropriate CAD4TB threshold. Delft Imaging - https://delft.care/wp-content/uploads/Choosing-the-appropriate-CAD4TB-threshold.pdf [↑](#footnote-ref-2)
